# T-cell redirecting therapies for B-cell non-Hodgkin lymphoma: recent progress and future directions

**DOI:** 10.3389/fonc.2023.1168622

**Published:** 2023-07-03

**Authors:** David A. Russler-Germain, Armin Ghobadi

**Affiliations:** ^1^ Division of Oncology, Washington University School of Medicine, St. Louis, MO, United States; ^2^ Siteman Cancer Center, Washington University School of Medicine, St. Louis, MO, United States

**Keywords:** bispecific, antibody, T cell, engager, lymphoma, immunotherapy

## Abstract

Several key advances in the treatment of B-cell non-Hodgkin lymphoma (B-NHL) over the past two decades have strategically exploited B-cell lineage markers suitable for targeting by immunotherapies. First, the addition of the anti-CD20 monoclonal antibody (mAb) rituximab to a range of standard therapies conferred remarkable outcomes improvements in diverse settings, perhaps most prominently an overall survival advantage in newly diagnosed diffuse large B-cell lymphoma (DLBCL). Subsequently, multiple chimeric antigen receptor (CAR) T-cell therapies targeting CD19 have revolutionized the treatment of relapsed/refractory (rel/ref) DLBCL and are active in other B-NHL subtypes as well. Most recently, the longstanding aspiration to exploit patients’ endogenous T-cells to combat lymphoma has been achieved via T-cell redirecting therapies such as bispecific antibodies (BsAbs) that incorporate dual targeting of a T-cell antigen such as CD3 plus a B-cell antigen such as CD19 or CD20 expressed by the tumor. These novel agents have demonstrated impressive activity as monotherapies in patients with heavily pre-treated, rel/ref B-NHL of a variety of subtypes. Now, myriad clinical trials are exploring combinations of T-cell redirectors with targeted therapies, antibody-drug conjugates, conventional chemotherapy, and even new immunotherapies. Here, we highlight key landmarks in the development of T-cell redirecting therapies for the treatment of B-NHL, emerging evidence and lessons from recent clinical trials, and exciting new directions in this arena.

## 1 Introduction

Despite the clinical and genetic heterogeneity of B-cell non-Hodgkin lymphoma (B-NHL), many of the same cell surface markers used by pathologists in the diagnosis and specific subtyping of lymphoma are also attractive targets for anti-cancer therapies of various modalities. CD20 is expressed at least to some degree on the majority of mature B-cell neoplasms (1). As a typically non-internalizing surface antigen, CD20 is remarkably well-suited for targeting by monoclonal antibodies (mAbs). Key approved agents are rituximab (2, 3) and more recently obinutuzumab (4–6) and ofatumumab (7, 8), which are immunoglobulin G (IgG) molecules. They intentionally possess active fragment crystallizable (Fc) regions that trigger complement-mediated cytotoxicity (CMC) and antibody-dependent cell-mediated cytotoxicity (ADCC), thus function as immunotherapies predominantly mediated by natural killer (NK) cells and macrophages.

The general concept of T-cell immunotherapy for hematopoietic malignancies is well-established and dates back over half a century to the advent of allogeneic hematopoietic cell transplantation, which seeks to optimize graft-versus-leukemia/lymphoma (GvL) activity while minimizing graft-versus-host (GvH) effects. T-cell immunotherapies for solid tumors were largely underwhelming until the past decade, during which there has been a rapid expansion of indications for immune checkpoint inhibitors (ICI) as monotherapies and in combinations with other drugs for the treatment of many different cancers (9). By comparison, the anti-PD-1 and anti-PD-L1 mAbs have unfortunately been relative disappointments in B-NHL despite impressive activity in classical Hodgkin lymphoma (10–13). Instead, two major alternative strategies have emerged from the ambition to apply T-cell immunotherapy to B-NHL, both of which exploit the well-established B-cell lineage surface markers to target autologous T-cells to tumors.

**Figure 1:**
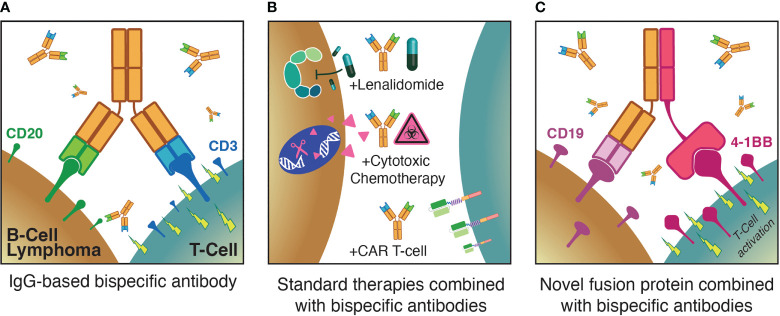
Three major themes for optimization of T-cell redirecting therapies for B-NHL: **(A)** Monotherapy anti-CD3xCD20 bispecific antibodies; **(B)** combinations of bispecific antibodies with established standard therapies; **(C)** augmenting bispecific antibodies with novel agents such as T-cell activating fusion proteins.

Chimeric antigen receptor (CAR) T-cell therapies are genetically engineered autologous products — the currently approved agents all target CD19, are highly active in diverse B-NHL subtypes and play important roles in the treatment of relapsed/refractory (rel/ref) aggressive B-NHL (14–18). However, CAR T-cell therapies suffer from substantial limitations with regard to the rate and severity of treatment-related toxicities including cytokine release syndrome (CRS), immune effector cell-associated neurotoxicity syndrome (ICANS), and cytopenias, prolonged referral-to-infusion times due to financial and production limitations, and deficient global access to these personalized immunotherapies (19–23). In contrast, off-the-shelf T-cell redirecting therapies have demonstrated impressive efficacy and safety profiles in early clinical trials over the past few years, with lower rates of CRS as well as minimal neurologic adverse events (AEs). As a result, the scalability of approaches such as BsAbs is poised to revolutionize the treatment landscape of B-NHL. Here, we first briefly summarize the initial application of T-cell redirecting therapy to B-NHL and highlight key lessons learned. We then discuss three pivotal themes regarding the ongoing optimization of T-cell redirecting therapies for B-NHL (**Figure 1**), describing recent clinical trial results that shed light on these issues. Finally, we discuss future directions in the field.

## 2 The opening chapter of T-cell redirecting therapy in B-NHL

The first off-the-shelf T-cell redirecting therapy studied in B-NHL was blinatumomab, which is a fusion protein comprised of two single-chain Fragment variable (scFv) components that target CD3 on the surface of patients’ endogenous T-cells and CD19 on the surface of malignant (and non-malignant) B-cells, coupled by a short non-immunogenic linker peptide (24). The resulting 55 kDa molecule is approximately one-third the size of a typical IgG molecule, which facilitated manufacturing based on its design, but unfortunately also led to pharmacokinetic properties that necessitated administration via multi-week continuous intravenous infusion (CIVI).

Blinatumomab was initially granted accelerated approval by the United States (US) Food and Drug Administration (FDA) in 2015 for the treatment of relapsed/refractory B-cell acute lymphoblastic leukemia (B-ALL) (25) based on a single-arm phase 2 trial in which 32% of patients receiving blinatumomab achieved a complete remission and the median duration of response (DOR) was 6.7 months (26). A subsequent phase 3 randomized controlled trial (RCT) confirmed the superiority of blinatumomab over standard chemotherapy in this setting (27). Since then, blinatumomab use has been expanded to the treatment of patients with B-ALL in remission with detectable measurable residual disease (MRD) (28), as well as for post-induction consolidation in B-ALL irrespective of MRD status (29).

Unfortunately, blinatumomab is associated with significant rates of CRS and ICANS (30, 31). In the phase 1 trial of blinatumomab in rel/ref B-NHL, among the 35 patients treated at the maximally tolerated dose (MTD) of 60 mg/m (2)/day, the overall response rate (ORR) was 69% with an impressive median DOR greater than one-year (32). However, neurologic AEs were dose limiting and the most frequent cause for early treatment discontinuation, with grade 3 events occurring in 22% of patients. Modified administration strategies were explored in a subsequent phase 2 study in rel/ref DLBCL, including step-up dosing and prophylactic dexamethasone, but an optimal approach to reach the target dose without early dropout was not achieved despite observing clear anti-tumor activity (33). This led to highly divergent interest in advancing blinatumomab for different CD19-expressing malignancies given risk-benefit tradeoffs that varied depending on the specific patient population and alternative treatment options.

These experiences with blinatumomab provided multiple essential lessons for the next generation of T-cell redirecting therapies. First, the requisite administration of blinatumomab via multi-week CIVI was largely a consequence of the linked dual scFv design that was perhaps the most reliably manufactured BsAb structure at the time. This inconvenience and the associated toxicities were deemed acceptable tradeoffs in rel/ref B-ALL given the aggressive nature of this disease and the comparable magnitude of logistical and toxicity burdens associated with alternative intensive treatments. Instead, more convenient, and possibly safer administration of a T-cell redirecting therapy could hopefully be achieved with the predictable kinetics of a full IgG BsAb structure. Generating asymmetric T-cell redirecting IgG molecules required novel molecular biology approaches, of which several have recently been developed and focus on production of custom engineered half-molecules that exclusively heterodimerize (34). Second, the struggles of blinatumomab dose optimization foreshadowed potentially similar difficulties with the next generation of T-cell redirecting therapies. In turn, as discussed below, the current spectrum of BsAbs in clinical development have required complex trial designs to explore diverse dosing approaches in disease-specific cohorts. Finally, the evidence of long-term responders after fixed-duration blinatumomab in rel/ref B-NHL (35) provided the necessary confidence to persevere with the advancement of T-cell redirecting therapies in B-NHL while concurrent successes were being seen with CAR T-cell therapies.

## 3 Theme #1: optimizing T-cell redirector monotherapy across indications?

**Figure 2:**
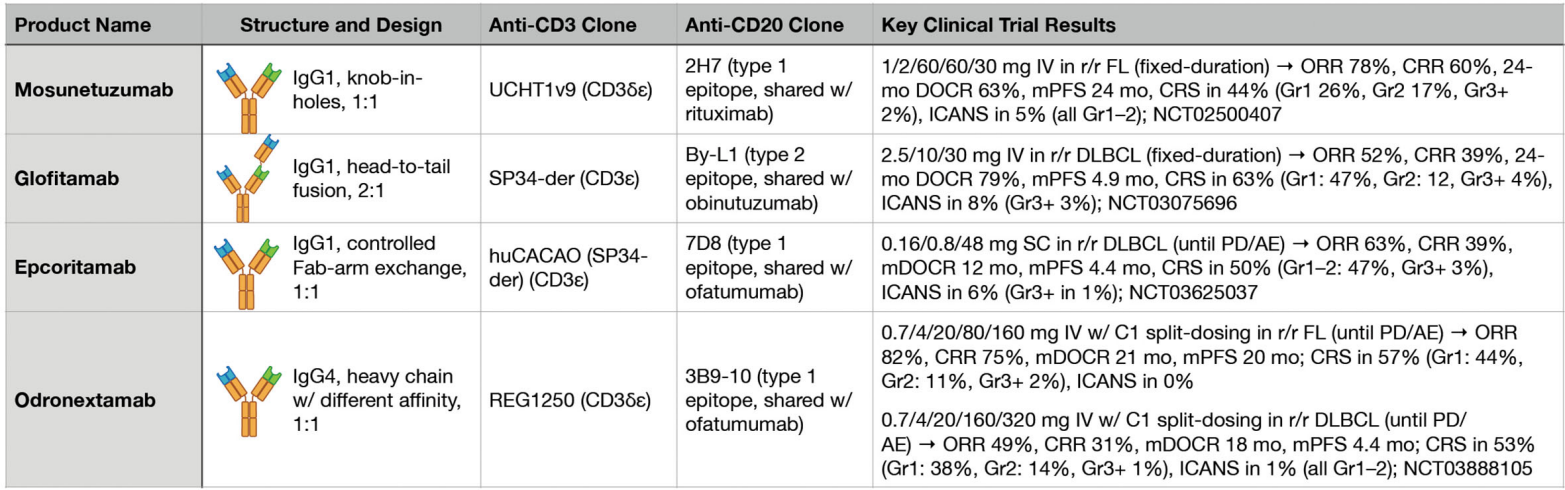
Key features and clinical trial results for four major anti-CD3xCD20 bispecific antibodies.

During the clinical development of T-cell redirecting therapies in B-NHL, perhaps the most fundamental issue being confronted has been the general question of, “How much is enough?” Broadly speaking, early phase clinical trials have had to address both dosing strategy and treatment duration in the context of the potency of the specific investigational agent. While imperfect, promising answers to this question have started to emerge based on results from multiple recent clinical trials of different BsAbs targeting CD3 and CD20 (**Figure 2**).

Mosunetuzumab is a first-in-class anti-CD3xCD20 BsAb that is a full-length, fully humanized IgG1 molecule with near-native antibody architecture constructed using ‘knobs-into-holes’ technology (36). The introduction of an N297G mutation results in a non-glycosylated heavy chain with abrogated FcγR binding. Initial pre-clinical studies demonstrated that mosunetuzumab was highly active in killing CD20-expressing B-cells, including primary patient leukemia and lymphoma cells both *in vitro* and *in vivo*. In non-human primates, mosunetuzumab depleted B-cells in the peripheral blood and lymphoid tissues at a single dose of 1 mg/kg while demonstrating pharmacokinetic properties similar to a conventional mAb. It also exhibited activity *in vitro* and *in vivo* in the presence of competing anti-CD20 mAbs, a key result given the high likelihood of patients with rel/ref B-NHL receiving mosunetuzumab having been previously treated with rituximab.

The phase 1 study of mosunetuzumab enrolled patients with rel/ref B-NHL expected to express CD20 (37). It included dose-escalation and dose-expansion cohorts with fixed and step-up dosing, as well as explored intravenous (IV) and subcutaneous (SC) administration strategies. For fixed dosing, mosunetuzumab was administered on day 1 of each 21-day cycle. For step-up dosing, mosunetuzumab was administered as low and intermediate step-up doses on days 1 and 8 of cycle 1, followed by the target dose on cycle 1 day 15 and on day 1 of each subsequent 21-day cycle. All patients received fixed-duration treatment, stopping after eight cycles if achieving a complete response (CR) or after 17 cycles if achieving a partial response (PR) or stable disease (SD) after eight cycles. Common adverse events (AEs) included CRS, leukopenia, hypophosphatemia, and fatigue. While rates of CRS of any grade were comparable between the initial fixed and step-up dosing cohorts at 24% and 27%, respectively, the step-up approach was favored due to shifting CRS from predominantly grade 2 to grade 1. To better characterize the safety and efficacy of mosunetuzumab, additional patients were treated in histology-specific interim expansion cohorts at each step-up dose level. The maximally tolerated dose (MTD) was not exceeded, and an initial IV recommended phase 2 dose (RP2D) of 1/2/13.5 mg step-up dosing for patients with rel/ref follicular lymphoma (FL) was preliminarily determined. However, based on additional data from dose-escalation and integrated exposure-response modeling, loading doses of 60 mg were suggested to be further along the plateau of the exposure-response curve for efficacy. In turn, an RP2D of 1/2/60 mg IV (cycle 1 days 1/8/15), 60 mg IV (cycle 2 day 1), and 30 mg IV (cycle 3 day 1 and beyond) was selected for further study in all histologies. However, key differences in responses were seen in indolent versus aggressive B-NHL. Across the step-up doses investigated, the ORRs were 66% versus 35% and the CR rates were 49% versus 19% in patients with indolent versus aggressive B-NHL, respectively.

Based on the results of the subsequent pivotal phase 2 study, mosunetuzumab was granted accelerated approval by the US FDA in December 2022 for the treatment of patients with rel/ref FL after two or more prior lines of therapy (38, 39). In this study, patients had previously received both an anti-CD20 mAb and alkylator chemotherapy. The study population was relatively high-risk: 52% of patients had experienced progression of disease within 24 months of initial therapy (POD24) and 69% were refractory to their prior therapy. Patients received mosunetuzumab 1/2/60/60/30 mg IV with step-up dosing during cycle 1, and fixed-duration treatment for all patients identical to the phase 1 study. Hospitalization for monitoring was not required based on the safety profile of mosunetuzumab seen in the phase 1 study. The ORR was 78% and the CR rate was 60%, with a median time to response 1.4 months and median time to CR of 3 months. Impressively, after a median follow-up of 28.3 months, the median DOR and duration of CR (DOCR) were both not reached. The 24-month DOCR was 63% and median progression-free survival (PFS) was 24 months. CRS of any grade was experienced by 44% of patients (grade 1: 26%, grade 2: 17%, grade 3: 1%, grade 4: 1%). CRS predominantly occurred after cycle 1 day 1 or day 15, and 8% of patients received tocilizumab for CRS management. Neurological adverse events deemed related to mosunetuzumab and consistent with ICANS occurred in five patients (all grade 1–2) and all episodes resolved; no aphasia, seizures, encephalopathy, or cerebral edema were reported.

In contrast to the 1:1 configuration of mosunetuzumab, glofitamab is a novel T-cell redirecting BsAb with a unique 2:1 configuration that enables bivalent binding to CD20 on B-cells and monovalent binding to CD3 on T-cells (40). The CD3-binding region is fused to one of the CD20-binding regions in a head-to-tail manner via a flexible linker, which confers improved target-effector cell recognition. As a result, glofitamab showed greater *in vitro* potency than anti-CD3xCD20 BsAbs with a 1:1 configuration, leading to enhanced anti-tumor activity in preclinical models of DLBCL (41). Furthermore, CD20 bivalency helped preserve this potency in the presence of competing anti-CD20 mAbs. In turn, the increased potency of glofitamab seen in pre-clinical development suggested it could outperform other BsAbs as a T-cell redirector monotherapy in rel/ref aggressive B-NHL, where mosunetuzumab exhibited only modest single-agent activity.

Glofitamab was initially studied in a dose-escalation and expansion phase 1 clinical trial in patients with rel/ref B-NHL expected to express CD20 after one or more prior lines of therapy and without an available life-extending treatment option (42). Fixed and step-up glofitamab dosing cohorts were explored, and all patients received a single 1,000 mg dose of obinutuzumab seven days before the first dose of glofitamab to deplete peripheral and tissue B-cells based on pre-clinical evidence suggesting an increased risk of CRS due to the high affinity of glofitamab for CD20. Following initial single-patient cohorts, 171 subsequent patients received at least one dose of glofitamab, of which 50% experienced CRS of any grade (grade 3 or higher: 4%). Two patients experienced transient grade 3 ICANS-like symptoms. The ORR was 54% and the CR rate was 37% across all doses and histologies, with an ORR of 66% and CR rate of 57% being observed in patients receiving the RP2D. While only a small subset of patients, nine of 14 patients with rel/ref aggressive B-NHL receiving the RP2D achieved a CR, which was highly promising.

Recent results from the pivotal phase 2 study of glofitamab in rel/ref DLBCL after two or more prior lines of therapy have solidified the connection between T-cell redirecting therapy design and potency, thus indicating that different BsAb agents in this class may be better suited for the treatment of indolent versus aggressive B-NHL subtypes (43). After an initial dose of obinutuzumab on cycle 1 day 1, glofitamab monotherapy was administered by step-up IV dosing on cycle 1 day 8 and 15 at 2.5 and 10 mg, respectively, followed by 30 mg target doses on day 1 of cycles 2 through 12 for fixed-duration therapy for all patients irrespective of depth of response. Efficacy data include an ORR of 52% and a CR rate of 39%, with a median PFS of 4.9 months. Deeper analysis of the durability of responses to glofitamab provides vital perspective: across all 291 patients in the phase 1 and 2 studies receiving glofitamab doses of at least 0.6 mg (with or without step-up dosing), the median DOCR was not reached and the 24-month DOCR was a remarkable 79% (median DOCR follow-up from first CR was 18 months) (44). From end-of-treatment (EOT), the median PFS for responding patients had not been reached and the 12-month PFS was 93%. Similarly impressive durability of responses was seen among the subset of patients receiving the RP2D.

Fixed-duration glofitamab has also shown impressive efficacy as a monotherapy in rel/ref mantle cell lymphoma (MCL) (45). The presence of circulating malignant B-cells was suspected to be a risk factor for CRS (46), thus given the higher rate of peripheral blood involvement in MCL than most other B-NHL subtypes, cycle 1 day 1 obinutuzumab doses of 1,000 and 2,000 mg were studied to further mitigate this risk. Identical to the rel/ref DLBCL setting, patients with rel/ref MCL received step-up glofitamab dosing on cycle 1 day 8 and 15, followed by 30 mg target doses on day 1 of cycles 2 through 12 for fixed-duration therapy. In this study of 37 patients, 27 were refractory to their last prior therapy, 24 had previously received a Bruton’s tyrosine kinase inhibitor (BTKi), and two had received prior CAR T-cell therapy. Across all patients, the ORR was 84% with a CR rate of 73%, and no significant differences were seen based on prior BTKi exposure or obinutuzumab dosing. While median follow-up was short at only eight months, the median DOCR was 10 months with no post-EOT progression observed by the time of the analysis in patients responding at EOT. Rates and severity of CRS appeared lower with escalation of obinutuzumab to 2,000 mg on cycle 1 day 1: 4/16 versus 2/21 patients experienced grade 3 or higher CRS, and 11/16 versus 6/21 patients required tocilizumab with the 1,000 and 2,000 mg doses of obinutuzumab, respectively. Neurologic AEs consistent with ICANS occurred in five patients (all grade 1–2).

Beyond the clear influence of T-cell redirecting therapy design on potency, recent clinical trials are also providing crucial insight into the consequences of T-cell redirecting therapy treatment duration. While mosunetuzumab and glofitamab were studied with fixed-duration approaches, other anti-CD3xCD20 agents have been studying using indefinite treatment. Odronextamab provides a useful comparison to mosunetuzumab and glofitamab in this context. Odronextamab is a hinge-stabilized, fully humanized heterodimeric IgG4-based BsAb with a unique common light chain design and no FcγRIII binding (47). After an initial phase 1 study (48), odronextamab has been advanced in histology-specific expansion phase 2 studies. Recent data from the rel/ref FL and rel/ref DLBCL cohorts have revealed how each T-cell redirecting therapy will need intricate clinical optimization to account for the CRS risk profile of each agent and to assess whether longer treatment durations confer meaningful clinical benefit to patients.

To date, the phase 2 study of odronextamab in rel/ref FL has enrolled a relatively high-risk cohort of 131 safety-evaluable patients after two or more prior lines of therapy including an anti-CD20 mAb and alkylator chemotherapy (49). Overall, 48% of patients had experienced POD24 and 71% were refractory to their last prior therapy, which are very similar to the mosunetuzumab phase 2 rel/ref FL population. Odronextamab was administered on 21-day cycles, with step-up doses during cycle 1, intermediate doses during cycles 2 through 4, and full doses from cycle 5 and beyond until progression or intolerance. While mosunetuzumab, glofitamab, and odronextamab trials all utilized prophylactic dexamethasone during early doses to mitigate the risk of CRS, odronextamab uniquely underwent further dose optimization during the phase 2 study. The final schema consisted of split-dosing during cycle 1 step-up (0.7 mg divided over days 1/2, 4 mg divided over days 8/9, and 20 mg divided over days 15/16), followed by 80 mg on days 1/8/15 of cycles 2 through 4, and 160 mg every 2 weeks thereafter until progression or excess toxicity. After a median follow-up of 22.4 months for the 121 efficacy-evaluable patients the ORR was 82% and the CR rate was 75% across the two split-dosing step-up schemas. Median DOR and DOCR were both 21 months, and 12-month PFS was 64% with a median PFS of 20 months. Of note, 12 of 131 patients experienced fatal infections, and 36 of 63 patients receiving the final 0.7/4/20 mg split-dosing step-up schema experienced CRS of any grade (grade 1: 28, grade 2: 7, grade 3: 1) of which 12 received tocilizumab. Ten patients experienced treatment-emergent AEs leading to discontinuation, and one episode of low-grade ICANS was observed.

In the phase 2 study of odronextamab in rel/ref DLBCL, patients had received two or more prior lines of therapy including an anti-CD20 mAb and alkylator chemotherapy (50). As in the rel/ref FL cohort, the specific split-dosing step-up approach was modified mid-study to reduce the risk of CRS. Odronextamab was similarly administered via split-dosing during cycle 1 step-up (0.7 mg divided over days 1/2, 4 mg divided over days 8/9, and 20 mg divided over days 15/16), followed by 160 mg on days 1/8/15 of cycles 2 through 4, and 320 mg every 2 weeks thereafter until progression or excess toxicity. Of the 140 safety-evaluable patients, 57% had primary refractory disease and 16% had previously undergone autologous stem cell transplantation (ASCT). Of the 130 efficacy-evaluable patients, the ORR was 49% and the CR rate was 31%. While not powered for specific subgroup analyses, the reported ORRs of 36% versus 67% for patients with or without primary refractory disease, respectively, as well as 38% versus 74% for patients with low versus high international prognostic index (IPI) stratification, respectively, suggest potential differential activity of odronextamab in patients with adverse features. Overall, the median PFS was 4.4 months, the median DOR was 10 months, and the median DOCR was 18 months. With the final split-dosing step-up approach, CRS of any grade was experienced by 39 of 73 patients (grade 1: 28, grade 2: 10, grade 3: 1), of which 19 received tocilizumab and one experienced low-grade ICANS.

## 4 Theme #2: monotherapy or combinations?

The initial clinical trials of multiple anti-CD3xCD20 T-cell redirecting therapies have revealed the incredible promise of this class of agents in the treatment of B-NHL, but whether they are best utilized as monotherapies or in combination with established treatments remains unclear. Dosing strategy and treatment duration are certainly factors that influence this decision, and recent studies of epcoritamab highlight many key lessons learned thus far.

Epcoritamab is a full-length IgG1 BsAb built on the DuoBody controlled antigen-binding fragment (Fab)-arm exchange platform targeting CD3 and CD20 (51). It retains normal neonatal Fc receptor binding resulting in a longer plasma half-life. The Fc domain of epcoritamab is modified to silence Fc-mediated effector functions. Compared with other anti-CD3xCD20 bispecific antibody constructs, epcoritamab showed significantly higher *in vitro* potency at lower doses, which translated into epcoritamab retaining its anti-tumor activity *in vivo* in the presence of anti-CD20 mAbs. SC administration of epcoritamab was supported by non-human primate studies, showing a similar degree of prolonged B-cell depletion with SC and IV administration. Importantly, SC administration also resulted in delayed and lower peak cytokine levels than IV administration, suggesting the SC route could potentially reduce the risk of severe CRS. To date, the clinical trial portfolio of epcoritamab combinations and monotherapy provides excellent perspective on the questions the field faces as T-cell redirecting agents move into early lines of therapy for many diseases.

Similar to other agents in this class, epcoritamab was initially studied in a dose-escalation phase 1/2 trial of patients with rel/ref CD20-positive B-NHL, with step-up priming and intermediate doses followed by full doses of epcoritamab administered SC in 28-day cycles (52). At initial publication, 73 patients were enrolled of which 68 received escalating full doses (0.0128–60 mg) of epcoritamab. With no DLTs being observed and the MTD not being reached, the full dose of 48 mg was identified as the RP2D. CRS was seen in 59% of patients (all grade 1 or 2). No treatment discontinuations occurred due to treatment-related AEs. The ORR was 68% in the 46 patients with rel/ref DLBCL, and the CR rate was 45% for the subset of these patients receiving full epcoritamab doses of 12–60 mg — at the 48 mg dose level, the ORR was 88% and the CR rate was 38%. Of ten patients with rel/ref FL receiving full epcoritamab doses of 0.76–48 mg, the ORR was 90% and the CR rate was 50%.

In the phase 1/2 expansion in patients with rel/ref CD20-positive large B-cell lymphoma after at least two prior lines of therapy, epcoritamab was administered SC in 28-day cycles once weekly for three step-up doses during cycle 1, followed by full doses weekly during cycles 2 and 3, every two weeks during cycles 4 through 9, and once every four weeks starting with cycle 10 until disease progression or unacceptable toxicity (53). As of early 2022, 157 patients had been treated, of which 61% had primary refractory disease and 39% had previously received CAR T-cell therapy. At a median follow-up of 10.7 months, the ORR was 63% and the CR rate was 39%. The median DOR was 12 months for all responders, whereas the median DOCR was not reached. CRS of any grade was experienced by 50% of patients (grade 1-2: 47%, grade 3: 3%). ICANS occurred in 6% of patients with one fatal event.

Epcoritamab as a monotherapy is also being explored in a phase 1/2 study in Richter’s Syndrome (RS), which is a major area of unmet need in B-NHL (54). The EPCORE CLL-1 trial provides the first standalone dataset for BsAb therapy in RS. Initial dose-escalation in CLL revealed no DLTs and the MTD was not reached. Similar to other settings as a monotherapy, epcoritamab was administered SC in 28-day cycles with weekly step-up doses in weeks 1-3 of cycle 1, then full doses once weekly cycles 2 through 3, once every 2 weeks during cycles 4 through 9, and once every 4 weeks starting with cycle 10 until disease progression or unacceptable toxicity. Inpatient monitoring was required for the first four doses. Patients had to be ineligible for or declined chemotherapy, and up to one prior line of therapy for RS was permitted. This was a high-risk population, with five of ten patients possessing a *TP53* mutation and two of ten possessing a *NOTCH1* mutation. Seven of ten patients had received prior CLL therapy (chemoimmunotherapy: 7, BCL2i: 5, BTKi: 5, CAR T-cells: 1). Five of ten patients had received prior RS therapy including R-CHOP, R-DHAP, and VR-EPOCH. Preliminary data from the ten-patient expansion in RS (all with underlying CLL with transformation to DLBCL histology) are promising despite short median follow-up of 4.9 months, with an ORR of 60% and a CR rate of 50%. A higher CRS risk was anticipated given the likelihood of circulating malignant cells in the peripheral blood compared to *de novo* DLBCL. Indeed, nine of ten patients experienced CRS of any grade (all grade 1 or 2), and predominantly with the first full dose on cycle 1 day 15. Seven patients required tocilizumab. Notably, six of ten patients required an epcoritamab dose delay due to treatment-emergent AEs, although no ICANS was reported.

With these monotherapy trial data as a backdrop, comparisons with preliminary data from four trials of epcoritamab combinations emphasize the context-dependent benefit and toxicity of T-cell redirecting therapies. In FL, epcoritamab is being combined with rituximab and lenalidomide in the frontline and rel/ref settings in separate arms of the ongoing EPCORE NHL-2 study (55, 56). For previously untreated patients with FL meeting Groupe d’Etude des Lymphomes Folliculaires (GELF) criteria for high tumor burden, SC epcoritamab was administered on 28-day cycles on a weekly basis during cycles 1 and 2, followed by every four weeks from cycle 3 and beyond up to a maximum of two years. After a median follow-up of 8.1 months, the ORR was 94% and the CR rate was 85%. Of the 41 patients evaluable for toxicity, 54% experienced CRS (all grade 1 or 2), predominantly after the first full dose on cycle 1 day 15. Four patients required tocilizumab, one episode of reversible grade 1 ICANS was observed, and there were two deaths from COVID-19.

In the rel/ref FL setting, patients with FL meeting GELF criteria also received SC epcoritamab up to two years in duration with standard rituximab and lenalidomide. This study accrued a relative high-risk population, with 42% of patients having experienced POD24. At median follow-up of 6.4 months, the ORR was 95% and the CR rate was 80%. Across all high-risk subgroups the CR rate was >65%, indicating deep responses irrespective of adverse risk factors. While the toxicity profile supported outpatient administration, non-trivial AEs were noted including 40% experiencing grade 3-4 neutropenia. Three of 76 patients experienced fatal treatment-emergent AEs (all deaths from COVID-19 infection). CRS of any grade was experienced by 43% of patients (grade 1: 25%, grade 2: 8%), and eight patients required tocilizumab. One episode of grade 1 ICANS was reported, which occurred on day 22 and lasted seven days.

These experiences in untreated and rel/ref FL of combining epcoritamab with standard immune-based therapies of rituximab (an anti-CD20 mAb) and lenalidomide (an oral immunomodulatory agent) highlight the potential for T-cell engaging therapies to pair well with other drugs without an apparent increased risk of CRS. Larger studies are needed to more precisely estimate the ORR and CR rates of these triplets compared to rituximab and lenalidomide alone in either setting. Longer follow-up is also necessary to know whether responding patients realize long-term benefit with more durable remissions from the addition of epcoritamab, especially considering the likely real increase in cytopenias and infection risk when T-cell redirecting therapies are combined with rituximab and lenalidomide in indolent lymphomas. The potential for patients to receive BsAb monotherapy after rituximab and lenalidomide in the rel/ref setting also influences interpretation of these combination studies.

Multi-agent cytotoxic chemotherapy is nearly universally combined with the anti-CD20 mAb rituximab in both frontline and salvage treatment of DLBCL and other aggressive B-NHL subtypes. The dominant mechanisms of action for rituximab are mediated by NK cells and macrophages, and these effects and the net clinical benefit remain robust even in the setting of concurrent administration of intensive, multi-agent cytotoxic chemotherapy (57). Several lines of emerging data from diverse areas of oncology suggest that T-cell immunotherapies can remain effective despite concurrent administration of chemotherapy that in theory could also be toxic to the T-cells expanding in response to the immunotherapy. Recently, multiple studies in classical Hodgkin lymphoma revealed synergy between the anti-PD-1 mAbs pembrolizumab and nivolumab with various multi-agent chemotherapy backbones including GVD (gemcitabine, vinorelbine, and liposomal doxorubicin) (58) and ICE (ifosfamide, carboplatin, and etoposide) (59), providing further evidence that concurrent administration of cytotoxic agents does not necessarily abrogate the benefits from T-cell immunotherapies. In turn, despite relying on the activity of immune effector cells potentially susceptible to cytotoxic chemotherapy, prior studies suggest novel T-cell redirecting therapies including BsAbs may also be potent partners with chemotherapy-based approaches for treatment of B-NHL. Indeed, data from ongoing trials of epcoritamab combined with chemotherapy indicate that these novel combinations may be additive (or even synergistic) instead of counterproductive.

In rel/ref DLBCL, SC epcoritamab has been combined with rituximab plus DHAX/C (dexamethasone, high dose cytarabine, and either oxaliplatin or carboplatin) as salvage prior to ASCT in a phase 1/2 study (60). After an initial eight patient dose-escalation phase, 21 patients have been treated in the expansion phase with the standard target dose of 48 mg. Patients received up to three cycles of R-DHAX/C. Epcoritamab was administered concurrently on a weekly basis during cycles 1 through 3, and patients without progression that did not proceed with ASCT could receive indefinite epcoritamab monotherapy until disease progression or unacceptable toxicity. This study enrolled a high-risk cohort, with 19/29 patients experiencing disease progression within 12 months of initial therapy and 5/29 having no response to frontline treatment. With median follow-up of 12.6 months and 27 efficacy evaluable patients, the ORR was 85% and the CR rate was 67%, which were preserved in the primary refractory subgroup. Overall, 16 patients proceeded with ASCT and five continued epcoritamab monotherapy after combination therapy. No progression post-ASCT has been seen to-date and the median PFS has not been reached. CRS was experienced by 41% of patients (all grade 1 or 2), while 21% of patients experienced grade 3 or 4 infections and one patient experienced grade 2 ICANS.

A similar question was asked in a separate cohort of patients with rel/ref CD20-positive DLBCL who failed or were ineligible for ASCT. In this setting, epcoritamab was administered with gemcitabine and oxaliplatin (GemOx) (61). Patients received GemOx every two weeks for up to four cycles, with concurrent epcoritamab until progression or unacceptable toxicity (dosed weekly during cycles 1 through 3, every 2 weeks from cycles 4 through 9, and every 4 weeks from cycle 10 and beyond). This study also enrolled a high-risk population, with 56% of patients having primary refractory disease. Of 25 efficacy-evaluable patients, the ORR was a remarkable 92% and the CR rate was 60%, including responses in patients relapsing after prior CAR T-cell therapy. CRS was seen in 70% of patients (all grade 1 or 2) and one episode of grade 3 ICANS was reported. Six patients experienced grade 5 AEs, however, with attribution to epcoritamab being possible in two patients.

Interpreting the response rates of epcoritamab combinations relative to historical comparisons suffers from limitations regarding selection bias in clinical trials as well as evolution of response criteria over the past two decades, among other issues. Nonetheless, in settings such as rel/ref DLBCL in unfit patients (e.g., epcoritamab + GemOx), the ORR of 92% drastically outperformed expectations. In contrast, single-arm studies of combinations of T-cell redirecting therapies with frontline B-NHL regimens will be much harder to interpret given the more limited ability to improve response rates and the long-term follow-up required to interpret durability of responses. Importantly, the primary objectives of such a study are assessment of safety and the ability to preserve chemotherapy dose intensity with the addition of a novel agent to a curative-intent backbone. Prior efforts, such as combining the BTKi ibrutinib to R-CHOP in the PHOENIX study (62), highlighted how treatment-emergent AEs in older patients can easily lead to compromised R-CHOP chemotherapy dosing, thus undermining any potential mechanistic benefit from the addition of the targeted therapy. In the PHOENIX study, the subgroup of patients under 60-years-of-age better tolerated the addition of ibrutinib to R-CHOP and received higher cumulative chemotherapy doses, leading to a hypothesis-generating overall survival (OS) signal in this subgroup.

The ongoing phase 1 trial of glofitamab plus R-CHOP in untreated DLBCL is an excellent example of these dilemmas. In this study, patients receive R-CHOP alone during cycle 1, glofitamab step-up dosing occurs on days 8 and 15 of cycle 2 of R-CHOP, and then target-doses of glofitamab are administered concurrently with day 1 of R-CHOP from cycle 3 and beyond (for a maximum 6 to 8 total cycles) (63). Based on preliminary data, chemotherapy dose intensities were preserved, with >90% of intended cyclophosphamide and doxorubicin doses being received by 93% and 95% of patients, respectively. To date, of the 53 patients on the study receiving glofitamab in addition to R-CHOP, the ORR was 87% and the CR rate was 76%. Twelve patients required dose modifications or interruptions to glofitamab. Interestingly, only six of 56 safety-evaluable patients experienced any grade CRS (grade 1: 4, grade 2: 2) and two patients required tocilizumab. No glofitamab-related neurologic AEs potentially consistent with ICANS were reported.

## 5 Theme #3: novel targets, designs, or partners?

Beyond the four anti-CD3xCD20 BsAbs furthest in clinical develop as detailed above, myriad other T-cell redirecting therapy approaches are in pre-clinical and clinical development. These range from targeting alternative antigens, using unique molecular designs, or finding innovative ways to augment the activity of the anti-CD3xCD20 agents outlined earlier, among other approaches.

First, it is unclear whether further optimization of the anti-CD3xCD20 BsAb design will be clinically beneficial. Plamotamab is a new IgG-based agent being studied in a phase 1 dose-escalation trial in rel/ref DLBCL or FL (64). Given a preliminary ORR of 47% in rel/ref DLBCL at the RP2D, it remains to be seen whether this agent can distinguish itself from others in the class unless a particularly advantageous safety profile is observed, or the dosing schema and treatment duration are uniquely favorable. In contrast, IGM-2323 is a first-in-class IgM-based BsAb, which possesses ten binding units for CD20 (65). While this theoretically would enhance its affinity for target cells, the larger IgM design also impacts the drug’s pharmacokinetic profile. Data from a 2021 presentation describe a 35% ORR in 23 efficacy-evaluable patients with rel/ref indolent or aggressive B-NHL, as well as cases of pseudo-progression.

Not deterred by the ups and downs during clinical development of the first anti-CD3xCD19 BsAb blinatumomab, TNB-486 is a novel anti-CD3xCD19 BsAb in early phase clinical development (66). It is a second-generation T-cell engager with an asymmetric, fully human IgG4 ‘rational’ design to maintain high efficacy while reducing toxicity due to a unique anti-CD3 domain aimed at reducing CRS (67). It also has a high-affinity, heavy-chain only anti-CD19 domain as well as a silenced Fc tail to prevent non-specific binding or ADCC. In the phase 1 study of patients with rel/ref CD19-positive B-NHL after two or more prior lines of therapy, TNB-486 was dosed every two weeks on 28-day cycles through cycle 6, followed by every four weeks thereafter for patients in CR. Hospitalization was required on days 1 and 15 of cycle 1 only. Fixed and step-up dosing cohorts are enrolling, with 30 patients having received at least one dose. Of these, seven were CD20-negative and seven had received prior CAR T-cell therapy. Based on 25 efficacy-evaluable patients, the ORR was 81% and the CR rate was 69% at doses of 2.4 mg or greater. No relapses post-CR have been observed, although all patients have been followed for less than two years. CRS of any grade was noted in 18 of 30 patients (grade 1: 11, grade 2: 6, grade 3: 1), all of which occurred during cycle 1 and rapidly resolved. Nine patients required tocilizumab. Of note, ICANS was experienced by 10 of 30 patients including four grade 3 episodes – of these, three patients were re-challenged with TNB-486 without recurrence of ICANS. It is speculated that the higher rate of ICANS with this agent is related to the CD19-targeting based on comparisons between experiences with blinatumomab, anti-CD19 CAR T-cells, and CD20-targeting BsAbs. Further, it is proposed that given the robust targeting of CD20 in B-NHL, a BsAb targeting CD19 could help address CD20 antigen loss. However, the theoretical potential for a CD19-targeting BsAb to interfere with future responses to anti-CD19 CAR T-cell therapies may limit the use of this agent, if approved, to CD19-positive post-CAR T-cell relapses, or in resource-limited settings where CAR-T cells are not financially viable at the present moment. Interestingly, there are ongoing clinical trials exploring different sequencing of anti-CD19 CAR T-cell therapy and anti-CD3xCD20 BsAb treatment in rel/ref aggressive B-NHL (e.g., NCT04703686, NCT04889716, NCT05260957).

Next, studies of trispecific antibody designs for T-cell redirecting therapies are now being initiated. Notable examples include JNJ-80948543, which is a fully human IgG1 with an anti-CD3e scFv, anti-CD20 scFv, and anti-CD79b Fab (68). It has a silent Fc and exhibits low affinity binding to CD3 that results in low *in vitro* cytokine production. PIT565 is an anti-CD2xCD3xCD19 agent (69). Interestingly, the CD2 ligand is CD58, loss of which confers resistance to CAR T-cell therapies (70, 71); synthetic activation of CD2 via binding of this trispecific molecule is hypothesized to address this issue. Finally, anti-CD3xCD19xCD22 molecules are in pre-clinical development (72).

Beyond canonical B-cell surface antigens of CD19, CD20, and CD22, T-cell redirecting therapies exploiting novel targets include NVG-111, a first-in-class, humanized, tandem scFv, anti-CD3xROR1 bispecific T-cell engager (73). It is being studied in a dose-escalation phase 1 trial of patients with rel/ref CLL or MCL after two or more prior lines of therapy. Two DLTs were noted within the first ten patients, including one episode of grade 3 ICANS. Four of ten patients experienced CRS (all grade 1 or 2), and 66% of evaluable patients had objective responses including two MRD negative CRs. CD1 is also expressed on CLL cells (in addition to expression in multiple myeloma and acute myeloid leukemia), and LAVA-051 is a humanized bispecific single domain (VHH) antibody that engages CD1d and the Vd2-TCR chain of T-cells to activate innate-like T-cell populations (74). Of the first 12 patients receiving LAVA-051 in a phase 1 dose-finding study, no CRS or ICANS was observed, with preliminary efficacy data anticipated within the next year.

CD30 is another antigen highly expressed in multiple hematologic malignancies, most notably classical Hodgkin lymphoma (cHL) and anaplastic large cell lymphoma (ALCL). Brentuximab vedotin (BV) is an antibody-drug conjugate (ADC) targeting CD30 approved for the treatment of cHL and T cell lymphoma (75–79). DuoBody-CD3xCD30 is an Fc-silenced, IgG1 BsAb in preclinical development with EC50 values in the sub-nanomolar range based on *in vitro* studies (80). Also targeting CD30 is AFM13, which is a tetravalent anti-CD16xCD30 BsAb that activates NK cells and macrophages via FcgRIII (81, 82). It is assembled via homodimerization of a single polypeptide in a head-to-tail fashion through noncovalent interactions of the immunoglobulin heavy (VH) and light (VL) variable chains of the constituting domains. Three of 26 evaluable patients achieved PR and 13 achieved SD in the phase 1 dose-escalation study of AFM13 as a monotherapy in rel/ref cHL (83). The pharmacokinetic profile of AFM13 necessitated significant dosing optimization the phase 2 study, exploring three-times-per-week dosing, weekly dosing, and weekly CIVI (84). A disappointing ORR of 16.7% was observed, leading to premature closure of the study. A parallel phase 1b study of AFM13 plus pembrolizumab in rel/ref cHL showed a more promising ORR of 83% (85), although the specific contribution of AFM13 is unclear and ICIs such as pembrolizumab are now more commonly combined with cytotoxic chemotherapy or BV in the treatment of patients with cHL. Ongoing early phase studies of AFM13 combined with cord-blood or cytokine-activated NK cellular immunotherapies have shown responses as well (86, 87).

Lastly, beyond T-cell redirecting therapies as monotherapies or in combination with established treatments, there is considerable excitement regarding efforts to boost the intrinsic activity of T-cell redirectors with the addition of novel immunotherapeutics. Specifically, one strategy is to mimic properties of CAR T-cells, which have both CD3z activation and either CD28 or 4-1BB co-stimulatory signaling domains (88). Thus, could anti-CD3xCD20 agents be augmented via induction of costimulatory signaling? Aiming to accomplish this, RG6333 is an anti-CD19xCD28 BsAb (89), and RG6076 is a monovalent anti-CD19 IgG1 molecule to which trimeric human 4-1BBL is fused (90) — both molecules have silent Fc domains and aim to provide a strong but safe ‘signal II’ to activated T cells. Interestingly, while prior studies of CD28 superagonists led to profound toxicity due to broad T-cell activation, BsAbs targeting a tumor-specific antigen (e.g., CD19) and CD28 show limited toxicity (and minimal activity) as monotherapies in humanized mouse and primate models (91). Both RG6333 and RG6076 are being studied in combination with glofitamab in phase 1 clinical trials (NCT05219513, NCT04077723).

## 6 Discussion

Despite the many limitations of cross-trial comparisons, key lessons can still be learned from an integrative analysis of the phase 2 experiences for mosunetuzumab, glofitamab, odronextamab, and epcoritamab. Clinical trials of these agents have all converged on a CRS mitigation strategy that incorporates step-up dosing with pre-treatment prophylactic dexamethasone during early doses, which appears to be successful at helping patients safely reach the target dose. However, it is clear that unique properties of these agents, despite their common anti-CD3xCD20 design, confer pharmacokinetic and pharmacodynamic properties that drive distinctive on-target activity and CRS risk profiles. Specific agents such as mosunetuzumab and glofitamab appear well-suited for use as monotherapies in the rel/ref indolent and aggressive B-NHL subtypes, respectively. Their less frequent administration over a fixed-duration treatment course is attractive in terms of minimizing patient time toxicity (92). One would hypothesize that this may also lead to a reduced risk of infection compared to indefinite treatment approaches such as with odronextamab despite its promising ORRs and CR rates. Further, strategies using retreatment (permitted in mosunetuzumab and glofitamab studies) as opposed to indefinite treatment may actually provide a net beneficial based on preliminary work suggesting intermittent dosing of T-cell redirecting therapies may help reduce T-cell exhaustion and facilitate improved tumor control (93). Hopefully, novel response assessment approaches, such using as circulating tumor DNA (ctDNA) sequencing for MRD assessment (94, 95), can guide future BsAb administration.

From a practical perspective, the current and likely future absence of direct head-to-head comparisons between these BsAbs means cross-trial comparisons and non-randomized real-world analyses are inevitable as physicians seek to determine the ideal balance between response rate, depth of response, durability of response, and both short- and long-term toxicities for individual patients who are candidates for a BsAb. Clinical trial data available to-date suggest that indefinite BsAb treatment does not clearly outperform fixed-duration therapy, and the convenience of BsAb administration (e.g., IV versus SC, frequency of drug administration) may be a major factor in selecting an agent for a specific patient. Additionally, once T-cell redirecting therapies become widely available, the ideal sequencing of anti-CD3xCD20 BsAbs with anti-CD19 CAR T-cell therapy will need to be determined, with unique answers likely existing for different lymphoma subtypes. For example, with BsAbs likely to first be approved for third-line therapy or later lines, BsAbs will be well-suited for off-the-shelf treatment of the >50% of patients with rel/ref DLBCL in whom second-line CAR T-cell therapy fails. In some settings, use of BsAb prior to CAR T-cells due to cellular therapy access issues will likely be very reasonable depending on the circumstances. In rel/ref FL, both the more indolent nature of the disease and the older patient population would suggest BsAbs will be used prior to CAR T-cells except in rare circumstances (e.g., younger patients with high-risk rel/ref FL seeking a briefer treatment course that may have curative potential).

Next, are T-cell redirecting therapies best used as monotherapies or in combinations? To date, studies of the anti-CD3xCD20 BsAbs have shown impressive response rates when used as single-agents, and in many cases CRs from BsAbs can be durable even after cessation of treatment, such as with mosunetuzumab and glofitamab. With aggressive B-NHLs often treated with curative intent in the first two or three lines of therapy, it is appealing to consider whether adding a T-cell redirecting therapy to standard first-, second-, or third-line treatment approaches could increase the curative fraction. It appears that combining these BsAbs with either cytotoxic chemotherapy or an immunomodulatory agent such as lenalidomide can be safe and effective, but larger and ideally randomized studies to clarify these issues more formally will be crucial. Preliminary response data for epcoritamab combinations, for example, are extremely promising. However, the benefits of the BsAb comes at the expense of additional, albeit variable, early (e.g., CRS and ICANS) and intermediate/late (e.g., infections) toxicities that are differentially tolerated in unique B-NHL subtypes and lines of therapy. It remains an open question what the optimal duration of T-cell redirecting therapy is when combined with standard backbones such as rituximab plus lenalidomide in newly diagnosed or rel/ref FL. Hopefully, longer clinical trial follow-up and correlative studies can shed light on whether briefer or intermittent BsAb dosing would be better tolerated but equally effective when used to augment established approaches in low-grade lymphomas, especially considering the activity and availability of BsAb monotherapies in later lines of treatment. When BsAbs have been combined with cytotoxic regimens in aggressive B-NHL, the risk of severe CRS appears low and the potential for a shorter course of BsAb therapy may help attenuate the additional risks of the resulting profound B-cell depletion. The durability of some remissions seen with fixed-duration T-cell redirecting monotherapy may foreshadow the ability of these agents to increase the curative fraction of patients with DLBCL treated in either the frontline or rel/ref settings, but this remains to be proven with longer follow-up of ongoing studies and likely larger RCTs in the future. The vast BsAb clinical trial portfolio even includes recently initiated clinical trials using MRD assessment via ctDNA sequencing as a method to influence treatment decisions, such as adding glofitamab to frontline R-CHOP in patients with detectable MRD mid-treatment (NCT04980222). Response-adapted approaches may strike the best compromise between integration of T-cell redirecting therapies into earlier lines of therapy while not exposing all patients to the additional toxicity associated with this class of agents.

Finally, what does the future hold regarding T-cell redirector targets, designs, and augmentation? Several non-canonical B-cell antigens such as ROR1 and CD1 are being targeted by novel BsAbs. Non-IgG molecules (e.g., IgM) and trispecific designs are also being explored. The addition of a second immunotherapeutic agent that triggers T-cell costimulatory signaling (e.g., CD28, 4-1BB) to anti-CD3xCD20 BsAbs was motivated in part by the successes of CAR T-cell therapy in B-NHL. The lymphoma community eagerly awaits the initial data from these clinical trials, as it will likely be difficult to predict the success of the next generation of T-cell redirecting therapies. The toxicities associated with specific immunotherapy targets can be unpredictable, such as the higher risk of ICANS in CD19-targeting therapies than CD20-targeting therapies, and even the unique neurocognitive and hypokinetic movement disorder with features of parkinsonism after BCMA-targeting CAR T-cell therapy in multiple myeloma (96). In turn, careful clinical trial design and execution will be required as the T-cell redirector class expands. Overall, the recent successes seen in early phase clinical trials of anti-CD3xCD20 BsAbs indicate that the future of B-NHL will surely involve T-cell redirecting therapies (97, 98).

## 7 Conclusions

T-cell redirecting therapy for the treatment of rel/ref B-NHL showed promise in the initial phase 1 studies of the anti-CD3xCD19 agent blinatumomab but struggles with dosing optimization to mitigate toxicity motivated alternative strategies. These included development of full bispecific IgG molecules as well as pivoting to targeting CD20. The subsequent phase 1 and 2 clinical trials of four anti-CD3xCD20 BsAbs (mosunetuzumab, glofitamab, odronextamab, and epcoritamab) have since shown T-cell redirecting therapy in B-NHL can be largely safe and effective. Answers to many fundamental questions regarding how to administer this novel class of drugs have derived from these studies, including the favorable outcomes from step-up dosing and prophylactic dexamethasone premedication during early cycles of treatment. The impressive anti-tumor activity and manageable toxicity profile of these four anti-CD3xCD20 BsAbs now open additional questions regarding their optimal integration into B-NHL treatment paradigms.

## Author contributions

DR-G and AG conceived of the manuscript, performed the research, and wrote the manuscript. All authors contributed to the article and approved the submitted version.
